# Long non-coding RNA expression profile in minor salivary gland of primary Sjögren’s syndrome

**DOI:** 10.1186/s13075-016-1005-2

**Published:** 2016-05-17

**Authors:** Huan Shi, Ningning Cao, Yiping Pu, Lisong Xie, Lingyan Zheng, Chuangqi Yu

**Affiliations:** Department of Oral Surgery, Affiliated Shanghai Ninth People’s Hospital, Shanghai Jiao Tong University School of Medicine, Shanghai Key Laboratory of Stomatology, Shanghai, China

**Keywords:** Long noncoding RNA, Sjögren’s syndrome, Expression profile, Pathogenesis

## Abstract

**Background:**

To examine the roles of long noncoding RNAs (lncRNAs) in the regulation of primary Sjögren’s syndrome (pSS) and reveal the expression profile of lncRNAs in labial salivary glands (LSGs) in pSS patients.

**Method:**

The expression of 63,431 lncRNAs and 39,887 mRNAs were determined in the LSG of four pSS patients and four healthy controls using microarray experiments. Validation was performed in 30 pSS patients and 16 controls using real-time PCR. LncRNA-mRNA co-expression and gene-pathway networks were constructed using bioinformatics software.

**Result:**

A total of 1243 lncRNAs (upregulated: 890, downregulated: 353) and 1457 mRNAs (upregulated: 1141, downregulated: 316) were differentially expressed in the LSGs of pSS patients (fold change >2, *P* <0.05). Eight of these lncRNAs were validated using real-time PCR. ENST00000420219.1 (3.13-fold), ENST00000455309.1 (2.51-fold), n336161 (2.45-fold), NR_002712 (2.41-fold), ENST00000546086.1 (1.94-fold), Lnc-UTS2D-1:1 (1.79-fold), n340599 (1.69-fold), and TCONS_l2_00014794 (1.28-fold) were significantly upregulated in pSS. There were strong correlations between these lncRNAs and β2 microglobulin, disease course, erythrocyte sedimentation rate (ESR), rheumatoid factor (RF), IgA, IgM, visual analogue scale (VAS) of parotid swelling and VAS of dry eyes. Computational analyses revealed that 28 of the differentially expressed (DE) mRNAs were associated with eight DE lncRNAs involved in chemokine signaling pathways, the nuclear factor-kappa B (NF-κB) signaling pathway, and tumor necrosis factor (TNF) signaling pathway.

**Conclusions:**

Our study revealed the expression profile of lncRNAs in LSGs of pSS patients. Many novel lncRNA transcripts that play important roles in the pathogenesis of pSS were dysregulated in pSS. Therefore, this study will aid in the development of new diagnostic biomarkers and drug therapies.

**Electronic supplementary material:**

The online version of this article (doi:10.1186/s13075-016-1005-2) contains supplementary material, which is available to authorized users.

## Background

Primary Sjögren’s syndrome (pSS) is an autoimmune disease that is characterized by the dysfunction of exocrine glands, primarily the salivary and lachrymal glands, which results in dry mouth and eyes. The extensive infiltration of sensitized lymphocytes into target glands leads to the primary pathological manifestations of pSS. The etiology of pSS not clear, but previous studies have suggested that hereditary, hormonal, and environmental factors play crucial roles in the onset and progression of pSS [1]. Activation of the innate/adaptive immune system is the first line of defense against infections and damaged tissues. However, aberrantly activated inflammatory processes underlie autoimmune disease. Persistent perturbations of these inflammatory pathways are detrimental to the host and eventually result in disease conditions, including rheumatoid arthritis (RA), cardiovascular disease, and cancer [2–4]. Therefore, inflammatory signaling pathways require strict regulation at the transcriptional and posttranscriptional levels. Recent studies have demonstrated that noncoding RNAs, especially microRNAs, play crucial roles in the regulation of inflammatory signaling pathways [5, 6]. The roles of long noncoding RNAs (lncRNAs) as novel regulators of these pathways have emerged in more recent years. LncRNAs are a newly discovered class of regulatory molecules that are not translated into proteins and consist of more than 200 nucleotides. LncRNAs strongly affect a variety of biological processes in cells and organ systems. Several studies revealed the strong involvement of lncRNAs in the regulation of the immune response, including several pathways of innate immunity [7, 8]. LincRNA-Cox2 is one lncRNA that both negatively and positively regulates the expression of many important immune-related genes. This lncRNA mediates its repressive effects on interferon-stimulated genes via interactions with hnRNP-A/B and A2/B1 [9, 10]. Recent studies have also identified THRIL as a key regulator of tumor necrosis factor alpha (TNF-α) induction on Toll-like receptor (TLR) 1/2 signaling in human THP1 macrophages. Knockdown of THRIL downregulated the production of TNF-α mRNA [11]. Several other lncRNAs, such as NEAT [12, 13], lnc-IL7R [14], PACER [15], lnc-DC [16], IL1β-RBT46 [17] and AS-IL1α [18], control the innate immune response, immune cell development, and adaptive immunity. These studies indicate the crucial role of lncRNAs in the normal immune system. However, it is important to identify whether lncRNA dysfunction is involved in the pathogenesis of autoimmune diseases. Increasing evidence suggests that the dysregulation of lncRNAs plays an important role in autoimmune diseases, such as systemic lupus erythematosus (SLE), RA, type I diabetes mellitus (T1DM), and multiple sclerosis (MS) [19–24]. The present study analyzed the lncRNA expression profiles in labial salivary glands (LSGs) of pSS patients using lncRNA microarray to investigate the potential roles of lncRNAs in the pathogenesis of pSS. The results provide a new direction for the diagnosis and therapy of pSS.

## Methods

### Study subjects

Thirty patients diagnosed with pSS and 16 control subjects were recruited from the Department of Oral Surgery, Shanghai Ninth People’s Hospital, School of Medicine, Shanghai Jiao Tong University. All of the selected pSS patients fulfilled the American-European consensus group criteria for pSS. Specimens were collected during labial biopsy. No immunosuppressive treatment was administered to patients prior to diagnosis. Sixteen control subjects were diagnosed with labial gland mucocele and underwent mucocele excision. Normal labial glands adjacent to mucocele were collected during surgery. The absence of acute infections or systemic diseases was confirmed in all healthy donors. All specimens were immediately frozen in liquid nitrogen after resection and stored at −80 °C until RNA extraction. The discovery cohort was composed of eight patients mixed with pSS patients and control subjects for the screening of 63,431 lncRNA transcripts. The independent validation cohort consisted of 30 pSS patients and 16 normal samples. Table 1 summarizes the detailed demographic, clinical and laboratory characteristics of the 30 pSS patients. All healthy donors were females aged 25–65 years old. Consent was obtained from each participant prior to sample collection. The Ethics Committee, Faculty of Medicine, Shanghai Jiao Tong University approved this study.

**Table 1:** The detailed demographic, clinical, and laboratory characteristics of 30 pSS patients

Characteristic	pSS patient
Sex, no. male/female	0/30
Age, mean ± SD years	47.63 ± 13.50
Dry mouth, VAS. mean ± SD	6.57 ± 2.12
Dry eyes, VAS. mean ± SD	2.90 ± 2.95
Parotid swelling, VAS. mean ± SD	4.00 ± 2.40
Rose bengal score, no. +/−	20/10
Saxon test (g/2 min). mean ± SD	1.44 ± 0.62
Grading of labial salivary gland biopsies, no.	
Nonspecific chronic sialadenitis	1
Grade 1 (<50 periductal lymphocytes)	1
Grade 2 (>50 periductal lymphocytes)	
Nonsegregated	6
Segregated aggregates	6
Grade 3 (>50 periductal lymphocytes, with GC-like structures)	16
Ro (SSA), no. +/−	24/6
La (SSB), no. +/−	10/20
Anti-centromere antibodies (ACA), no. +/−	6/24
ESR (mm/hr). mean ± SD	21.77 ± 12.95
RF (IU/ml). mean ± SD	146.21 ± 195.12
IgG (g/L). mean ± SD	19.12 ± 4.10
IgA (g/L). mean ± SD	3.22 ± 0.84
IgE (IU/ml). mean ± SD	78.54 ± 68.92
IgM (g/L). mean ± SD	1.73 ± 0.74
Course of disease (month). mean ± SD	35.53 ± 26.04
CRP (mg/L). mean ± SD	4.46 ± 2.15
C3 (g/L). mean ± SD	1.10 ± 0.19
C4 (g/L). mean ± SD	0.26 ± 0.07
β2 microglobulin(mg/L). mean ± SD	2.80 ± 0.88

*pSS* primary Sjögren’s syndrome, *SD* standard deviation, *VAS* visual analogue scale, *ESR* erythrocyte sedimentation rate, RF rheumatoid factor, *Ig* immunoglobulin, *CRP* C-reactive protein, *C3* complement 3, *C4* complement 4

### Microarray

Total RNA was extracted from eight samples (four pSS and four control subjects) using TRIzol reagent (Life Technologies, Carlsbad, CA, USA). The RNeasy Mini Kit (Qiagen, GmBH, Hilden, Germany) was used to purify the total RNA according to the manufacturer’s recommendation. Purified total RNA was quantified using a NanoDrop 1000 (Thermo Fisher Scientific, Waltham, MA, USA). The assessment of RNA integrity was determined using RNA LabChip™ kits and an Agilent 2100 bioanalyzer (Agilent Technologies, Santa Clara, CA, USA). Only samples with 2100 RIN ≥7.0 and 28S/18S ≥0.7 were used.

Agilent SurePrint G3 microarray was used to investigate 63,431 lncRNAs and 39,887 mRNAs. Total RNA was amplified and labeled using a Low Input Quick Amp Labeling Kit, One-Color (Agilent Technologies, Santa Clara, CA, US). Labeled cRNA were purified using an RNeasy mini kit (Qiagen, GmBH, Hilden, Germany). The microarray hybridization was performed based on the manufacturer’s standard protocols (Agilent Technologies, Santa Clara, CA, US). Slides were washed in staining dishes and scanned. Raw data were normalized using a Quantile algorithm in Gene Spring Software 11.0 (Agilent Technologies, Santa Clara, CA, US).

### LncRNA-mRNA co-expression network

An lncRNA-mRNA co-expression network was built based on the normalized signal intensity of differentially expressed lncRNAs and mRNAs to explore the dysregulation of lncRNAs in pSS patients. A co-expression network of control subjects and pSS patients was established. Differentially expressed lncRNAs and mRNAs that met the criteria (*P* value <0.05, fold change >2 or <0.5) were selected. Correlations between lncRNA and lncRNA, lncRNA and mRNA, mRNA and mRNA were investigated using Pearson’s correlations. Only strong correlations (*P* <0.001) were drawn in these renderings. The importance of a gene in this network is reflected by degree. A gene with a large degree indicated that it was at a central position in the network and it shared closer relationships with more genes. The last step investigated genes that exhibited different degrees in pSS patients and control subjects.

### Screening of differentially expressed genes for validation

We screened differentially expressed (DE) lncRNAs for further validation using the following two approaches to validate the results of microarray experiments in an independent cohort and investigate the correlations between gene expression levels and clinical characteristics.

First, genes were evaluated based on the data revealed by microarray experiments using the following criteria: (a) the fold change of genes must be greater than fivefold compared to control subjects; (b) the signal value of the probes in each sample must be greater than seven; (c) the signal of the probes in each sample must be significantly different from the background signal; and (d) genes with lncRNA-mRNA repeated sequences and without information in databases were excluded.

Second, the degree of differentially expressed genes between pSS patients and control subjects was compared based on the co-expression network results. The top 30 genes with high different degrees and the qualified requirements for signal values were selected.

### Real-time PCR

Total RNA was extracted as described above. cDNA was synthesized from 0.5 μg RNA using the iScript cDNA synthesis kit (Bio-Rad Laboratories, Hercules, CA, USA). Real-time PCR was performed using an ABI Power SYBR Green PCR Master Mix (ABI, Foster City, CA, USA) and 7900 HT Sequence Detection System (ABI, Foster City, CA, USA). Real-time PCR was performed with a 5-ng cDNA template using the 2× SYBR Green PCR buffer and 10-μmol PCR primers in a total volume of 10 μl. Reactions were performed in 384-well PCR microplates. Additional file 1: Table S1 lists the primer sequences.

The expression of each lncRNA was represented as fold changes using the △△Ct method to obtain quantitative results. Differences between groups were analyzed using a two-tailed Mann-Whitney *U* test or unpaired *t* test, based on the homogeneity of variance. Spearman’s test was used for correlation studies. A value of *P* <0.05 was considered significant.

### Immunohistochemistry

Immunohistochemical staining to detect C-X-C chemokine receptor type 4 (CXCR4), CD19, CD21, Toll-like receptor 9 (TLR9) and intercellular cell adhesion molecule 1 (ICAM1) was performed on LSG biopsy sections as described previously [25]. CXCR4 (ab124824, Abcam, Cambridge, MA, USA), CD3 (ab699, Abcam, Cambridge, MA, USA), CD19 (ab134114, Abcam, Cambridge, MA, USA), CD21 (ab75985, Abcam Cambridge, MA, USA), ICAM1 (ab53013, Abcam, Cambridge, MA, USA), CD20 (ab78237, Abcam, Cambridge, MA, USA), and TLR9 (BA3861-1, Boster Biotechnology, Wuhan, China) antibodies were used in this experiment. Negative control staining was performed by replacing primary antibodies with PBS. Positive immunoreactivity appeared as a brown color. Double staining for CD3 and CD20 was used to analyze T/B cell segregation using the DouMaxvision™ double-stain system (KIT-9998, Maixin Biotechnology, Fuzhou, China). A scoring system was used to describe the results of double staining as described previously [26]. The images of grade 1 to 3 are shown in Additional file 2: Figure S3.

### EBV-encoded RNA (EBER) in situ hybridization

In situ hybridization was performed in LSG of 30 pSS patients using Epstein-Barr Virus ISH Detection Kit (Triplex International Biosciences, Fuzhou, China), according to the manufacturer’s protocol. Patients with lymphepithelioma were used as positive controls. The images of EBER-positive cells in representative ectopic lymphoid structures are shown in Additional file 3: Figure S4.

### Bioinformatics analysis

Aberrantly expressed lncRNAs and mRNAs with statistical significance were identified using Volcano Plot filtering. The threshold used to screen up- or downregulated RNAs was a fold change >2.0 (*P* <0.05). Hierarchical clustering was performed in Cluster 3.0, and heat maps were generated in Java Treeview. The DE mRNAs or DE lncRNAs-related mRNAs were analyzed using pathway annotation and gene ontology (GO) functional enrichment in the Cytoscape 3.3 software. The gene-pathway network was also constructed using Cytoscape 3.3.

## Results

### Overview of aberrantly expressed lncRNAs and mRNAs in pSS

**Fig. 1:**
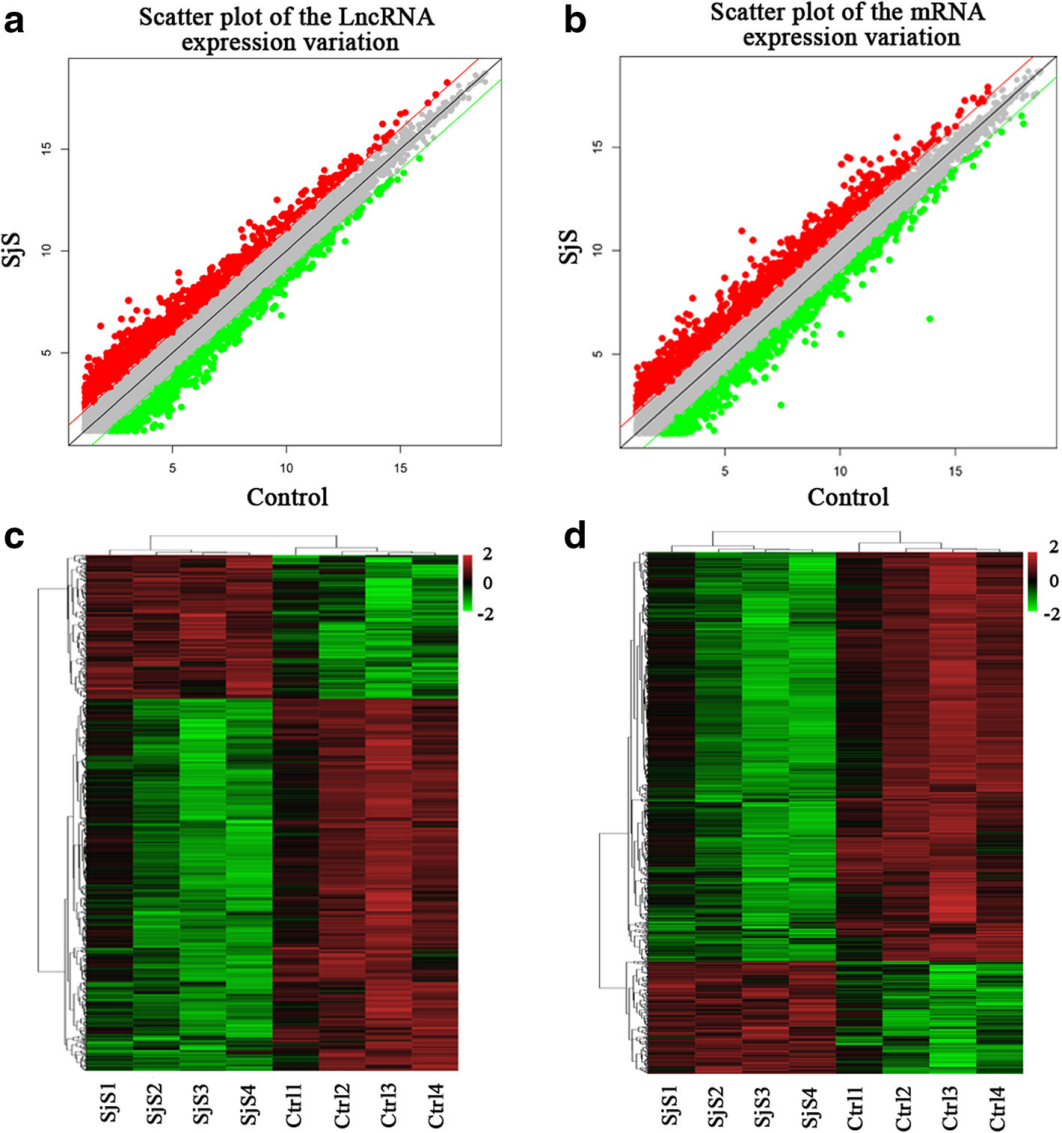
The expression profiling of lncRNA and mRNA in LSGs of pSS patients and healthy controls. **a**, **b** Scatter plot comparing global lncRNA or mRNA gene expression profiles between pSS and control subjects. *Green lines* and *red line* indicate twofold differences in either direction in lncRNA and mRNA expression. **c**, **d** Heat map showing hierarchical clustering of lncRNAs or mRNA with expression changes greater than twofold. *Red* and *green colors* represent up- and downregulated genes, respectively. *lncRNA* long noncoding RNA, *SjS* Sjögren’s syndrome

The expression levels of mRNAs and lncRNAs in four LSGs of pSS patients and paired control samples were analyzed using gene expression microarrays. The expression signatures of lncRNAs and mRNAs were reviewed using scatter plot and hierarchical clustering analyses. The scatter plots revealed that many lncRNAs and mRNAs were differentially expressed between pSS patients and control subjects (Fig. 1a, b). The heat maps of DE lncRNAs or mRNAs indicated the high level of concordance in pSS or control samples (Fig. 1c, d). The above-listed data indicated that changes in lncRNAs and mRNAs in LSGs were associated with the pathogenesis of pSS. Of the 63,431 lncRNAs and 39,887 mRNAs in the microarray, 1243 lncRNAs and 1457 mRNAs were significantly differentially expressed in the labial glands of pSS patients compared to control subjects (fold change >2, *P* <0.05). A total of 890 lncRNAs and 1141 mRNAs were upregulated, and 353 lncRNAs and 316 mRNAs were downregulated. Table 2 lists the 20 most up- and downregulated DE lncRNAs. Table 3 lists the 20 most up- and downregulated DE mRNAs. All of the data from the microarray trials are stored in the GEO database with accession number GSE76013.

**Table 2:** Top 20 significantly differential expressed lncRNAs between pSS patients and control subjects

Upregulated			Downregulated		
lncRNAs	*P* value	Fold change	lncRNAs	*P* value	Fold change
lnc-UTS2D-1:1	0.001	24.377	NR_026839.1	0.010	−7.135
NR_002712	0.018	20.057	TCONS_l2_00013113	0.007	−6.244
n341833	0.001	16.933	NR_026842.1	0.022	−5.943
lnc-MMP3-1:1	0.023	11.056	TCONS_l2_00004830	0.033	−5.319
lnc-LGALS14-1:2	0.000	9.526	NR_046443.1	0.033	−5.280
NR_034176.1	0.005	8.737	lnc-CYR61-2:1	0.008	−4.783
n333136	0.026	7.420	NR_037839	0.001	−4.760
n336199	0.031	6.893	NR_037839	0.022	−4.747
NR_073198.1	0.012	6.880	n333443	0.031	−4.633
lnc-MAGEA12-2:1	0.013	6.866	NR_026838	0.012	−4.358
lnc-ADAM2-1:1	0.020	6.853	NR_026838.1	0.025	−4.358
n337610	0.005	6.654	TCONS_l2_00019075	0.006	−4.350
n334829	0.007	6.628	ENST00000495382.1	0.014	−4.313
ENST00000577557.1	0.024	6.626	NR_036580	0.028	−4.308
XR_111691	0.010	6.482	lnc-OBP2B-2:1	0.011	−4.294
XR_111691	0.011	6.384	n333418	0.003	−4.256
lnc-KATNAL1-3:14	0.009	6.336	ENST00000508179.1	0.005	−4.180
n336575	0.017	6.215	n334464	0.004	−4.117
ENST00000578280.1	0.013	6.183	TCONS_l2_00010194	0.022	−4.005
lnc-BRD1-4:1	0.002	6.101	lnc-ZNF572-1:15	0.012	−3.962

*lncRNA* long noncoding RNA, *SjS* Sjögren’s syndrome

**Table 3:** Top 20 significantly differential expressed mRNAs between pSS patients and control subjects

Upregulated			Downregulated		
mRNAs	*P* value	Fold change	mRNAs	*P* value	Fold change
EGFL6	0.018	26.237	ITLN1	0.003	−104.642
SCGB1D2	0.020	17.197	TRH	0.001	−26.423
SCGB2A2	0.033	16.941	C4orf40	0.036	−9.826
CXCL13	0.018	16.911	LCE5A	0.001	−7.139
SCGB1D1	0.019	15.752	CYP2F1	0.013	−6.398
FCRL4	0.010	14.227	FXYD2	0.000	−6.070
MMP12	0.028	11.131	CADPS	0.002	−5.823
IDO1	0.024	9.307	ASTN1	0.038	−5.735
CD1A	0.007	8.942	CABS1	0.022	−5.372
CXCL11	0.018	8.175	GABRG3	0.005	−5.303
KLHDC7B	0.008	8.152	FGF12	0.039	−5.231
CXCL9	0.028	7.876	CSN3	0.008	−4.829
TIMD4	0.001	7.647	DACH2	0.048	−4.709
UTS2D	0.029	7.368	LEFTY1	0.027	−4.709
SLC26A4	0.014	7.037	NA	0.048	−4.652
ZBTB32	0.011	6.793	XLOC_002852	0.011	−4.508
TIMD4	0.004	6.746	NA	0.012	−4.375
EXOC3L4	0.003	6.281	PPP1R17	0.033	−4.332
IFNG	0.018	6.201	ABO	0.012	−4.272
CTLA4	0.011	6.177	SLC35G1	0.024	−4.215

*SjS* Sjögren’s syndrome

### Real-time PCR: lncRNA expression profiles and correlations with clinical characteristics

**Fig. 2:**
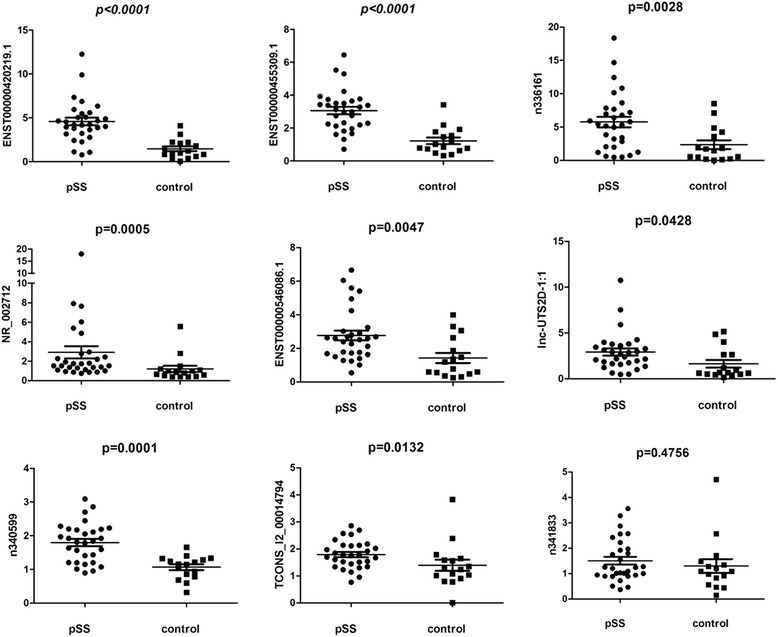
The expression level of nine lncRNAs in LSG of pSS patients and control subjects were identified using real-time PCR. We confirmed that eight lncRNAs were significantly upregulated, except n341833. ENST00000420219.1 (3.13-fold, *P* <0.0001), ENST00000455309.1(2.51-fold, *P* <0.0001), n336161(2.45-fold, *P* = 0.0028), NR_002712(2.41-fold, *P* = 0.0005), ENST00000546086.1 (1.94-fold, *P* = 0.0047), Lnc-UTS2D-1:1(1.79-fold, *P* = 0.0428), n340599(1.69-fold, *P* = 0.0001), and TCONS_l2_00014794(1.28-fold, *P* = 0.0132) were significantly increased in pSS. *pSS* primary Sjögren’s syndrome

**Fig. 3:**
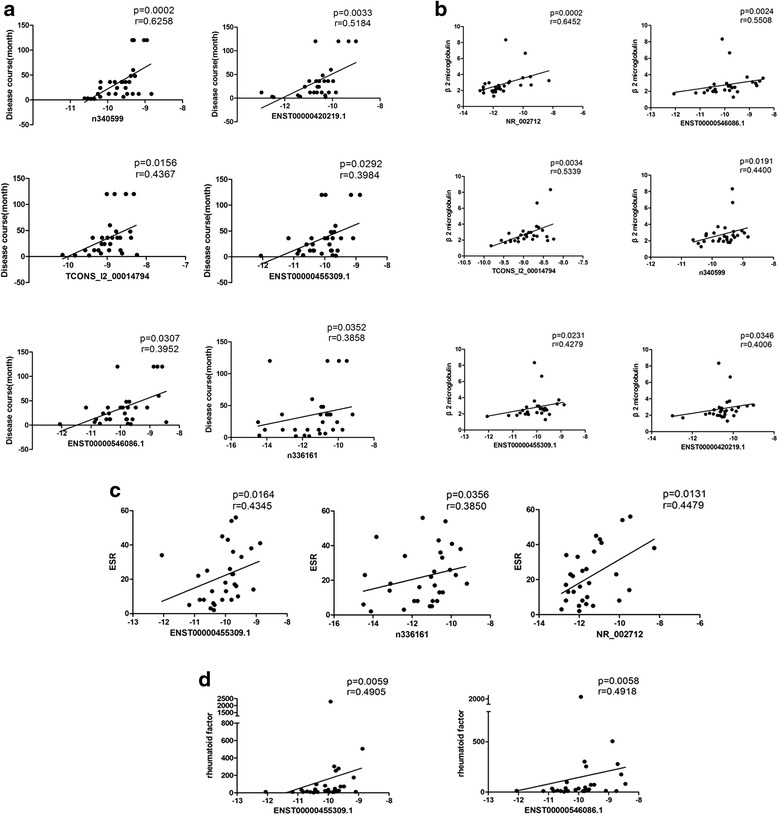
Correlation between lncRNAs and clinical characteristics in pSS. **a** The expression level of ENST00000420219.1, ENST00000455309.1, n340599, n336161, ENST00000546086.1, and TCONS_l2_00014794 were significantly associated with the course of disease. **b** The expression level of ENST00000420219.1, ENST00000455309.1, NR_002712, n340599, ENST00000546086.1, and TCONS_l2_00014794 were strongly associated with β2 microglobulin. **c** The expression level of ENST00000455309.1, NR_002712, and n336161 were associated with ESR. **d** The expression levels of ENST00000455309.1 and ENST00000546086.1 were significantly associated with rheumatoid factor (RF). *ESR* erythrocyte sedimentation rate

**Fig. 4:**
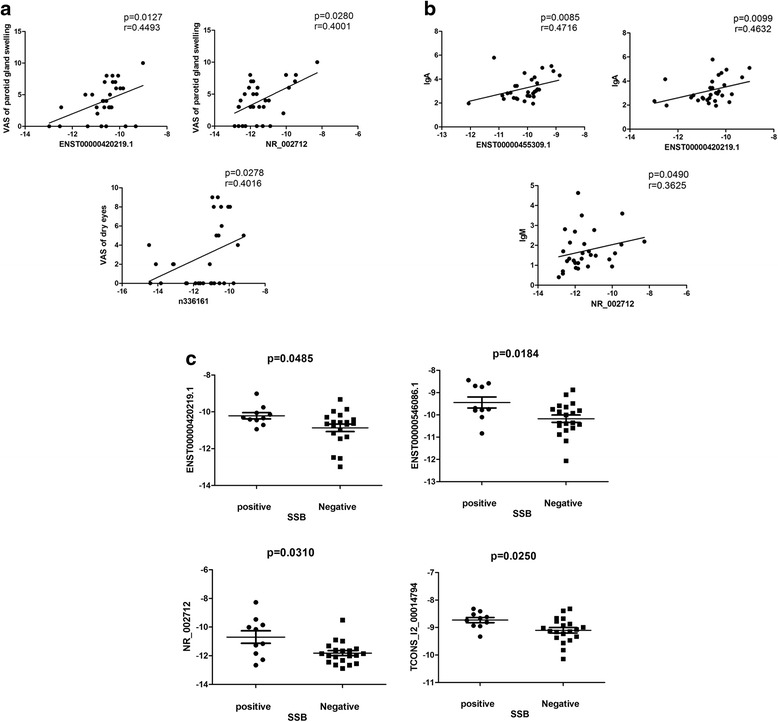
Correlation between DE lncRNAs and clinical characteristics in pSS. **a** The expression level of ENST00000420219.1 and NR_002712 were significantly correlated with VAS of parotid swelling, and the expression level of n336161 was associated with VAS of dry eyes. **b** The expression levels of ENST00000420219.1 and ENST00000455309.1 were significantly correlated with IgA, and the expression level of NR_002712 was associated with IgM. **c** The expression levels of ENST00000420219.1, NR_002712, ENST00000546086.1, and TCONS_l2_00014794 were significantly upregulated in SSB-positive patients compared with SSB-negative patients. *Ig* immunoglobulin, *VAS* visual analogue scale

Aberrantly expressed lncRNAs were identified in labial gland tissue samples from 30 pSS patients and 16 control subjects. The selection of representative lncRNAs for validation was based on the two approaches described in the Methods section. Nine lncRNAs were selected: NR_002712, n341833, lnc-UTS2D-1:1, TCONS_l2_00014794, n336161, ENST00000420219.1, ENST00000455309.1, n340599, and ENST00000546086.1. The results demonstrated that ENST00000420219.1 (3.13-fold), ENST00000455309.1 (2.51-fold), n336161 (2.45-fold), NR_002712 (2.41-fold), ENST00000546086.1 (1.94-fold), Lnc-UTS2D-1:1 (1.79-fold), n340599 (1.69-fold), and TCONS_l2_00014794 (1.28-fold) were significantly increased in pSS (Fig. 2). However, n341833 was not dysregulated in pSS patients. The next step was an analysis to determine whether any correlations existed between these DE lncRNA expression levels and the clinical characteristics. Figures 3a–d and 4a–b show that strong correlations were observed in eight pairs: (1) disease course with six lncRNA expression levels; (2) visual analogue scale (VAS) of parotid swelling with two lncRNAs; (3) VAS of dry eyes with one lncRNA; (4) β2 microglobulin with six lncRNAs; (5) erythrocyte sedimentation rate (ESR) with three lncRNAs; (6) rheumatoid factor (RF) with two lncRNAs; (7) immunoglobulin (Ig)A with two lncRNAs; and (8) IgM with one lncRNA. Four lncRNAs were significantly upregulated in SSB-positive patients compared to SSB-negative patients (Fig. 4c–d). No significant correlation existed between these DE lncRNAs and dry mouth, IgE, IgG, complement 3 (C3), complement 4 (C4), C-reactive protein (CRP) and grading of labial biopsy. Additional file 4: Table S2 shows the detailed correlation analysis results. A multivariate model was used to identify lncRNAs that correlated independently with disease characteristics. Additional file 5: Table S3 shows the results of this analysis.

### Functional prediction of DE mRNAs

**Fig. 5:**
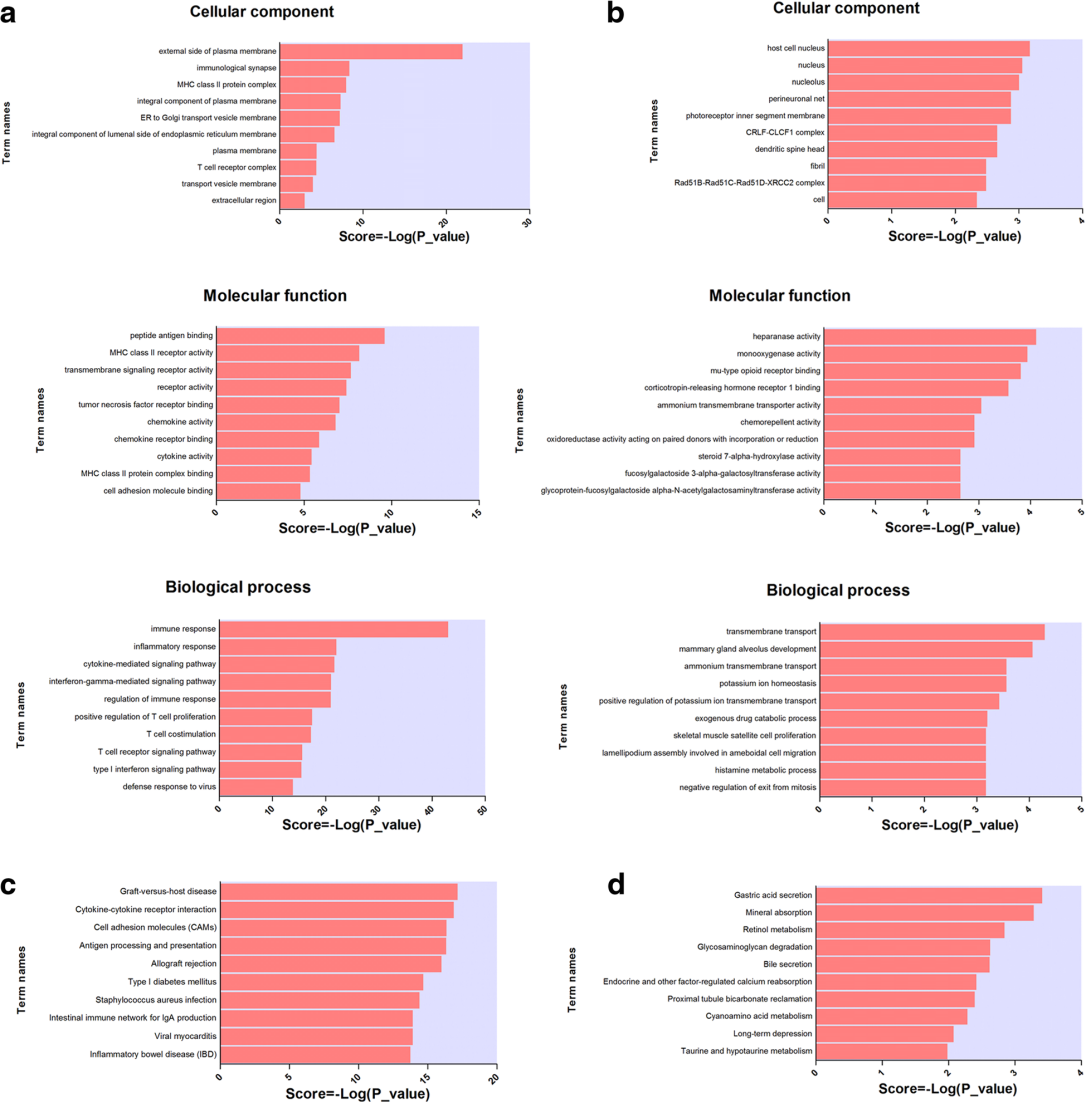
Biological functions of DE mRNAs (*P* <0.05, fold changes >2). **a** The significant molecular function, biological process, and cellular component of upregulated mRNAs. **b** The significant molecular function, biological process, and cellular component of downregulated mRNAs. **c** Significantly enriched pathways of upregulated mRNAs. **d** Significantly enriched pathways of downregulated mRNAs

GO and pathway analyses of the up- and downregulated genes in LSGs of pSS patients were performed to further examine the potential mechanism of pSS. The GO results indicated that the most significantly enriched cellular components of upregulated mRNAs in LSGs of pSS were the external side of the plasma membrane, immunological synapse, and MHC class II protein complex (Fig. 5a). The most significantly enriched molecular functions of upregulated mRNAs were peptide antigen binding, MHC class II receptor activity, and transmembrane signaling receptor activity (Fig. 5a). The most significantly enriched biological processes of upregulated mRNAs were immune response, inflammatory response, and cytokine-mediated signaling pathway (Fig. 5a). The most significantly enriched cellular components of downregulated mRNAs in LSGs of pSS were host cell nucleus, nucleus, and nucleolus (Fig. 5b). The most significantly enriched molecular functions of downregulated mRNAs were heparanase activity, monooxygenase activity, and mu-type opioid receptor binding (Fig. 5b). The most significantly enriched biological processes of downregulated mRNAs were transmembrane transport, mammary gland alveolus development, and potassium ion homeostasis (Fig. 5b). DE mRNAs were analyzed in the Kyoto Encyclopedia of Genes and Genomes (KEGG). The results revealed that the upregulated mRNAs in LSGs of pSS were significantly involved in graft-versus-host disease, cytokine-cytokine receptor interactions, and cell adhesion molecules (Fig. 5c). The downregulated mRNAs were significantly involved in gastric acid secretion, mineral absorption, and retinol metabolism (Fig. 5d).

### Relational analyses of lncRNAs and mRNAs

**Fig. 6:**
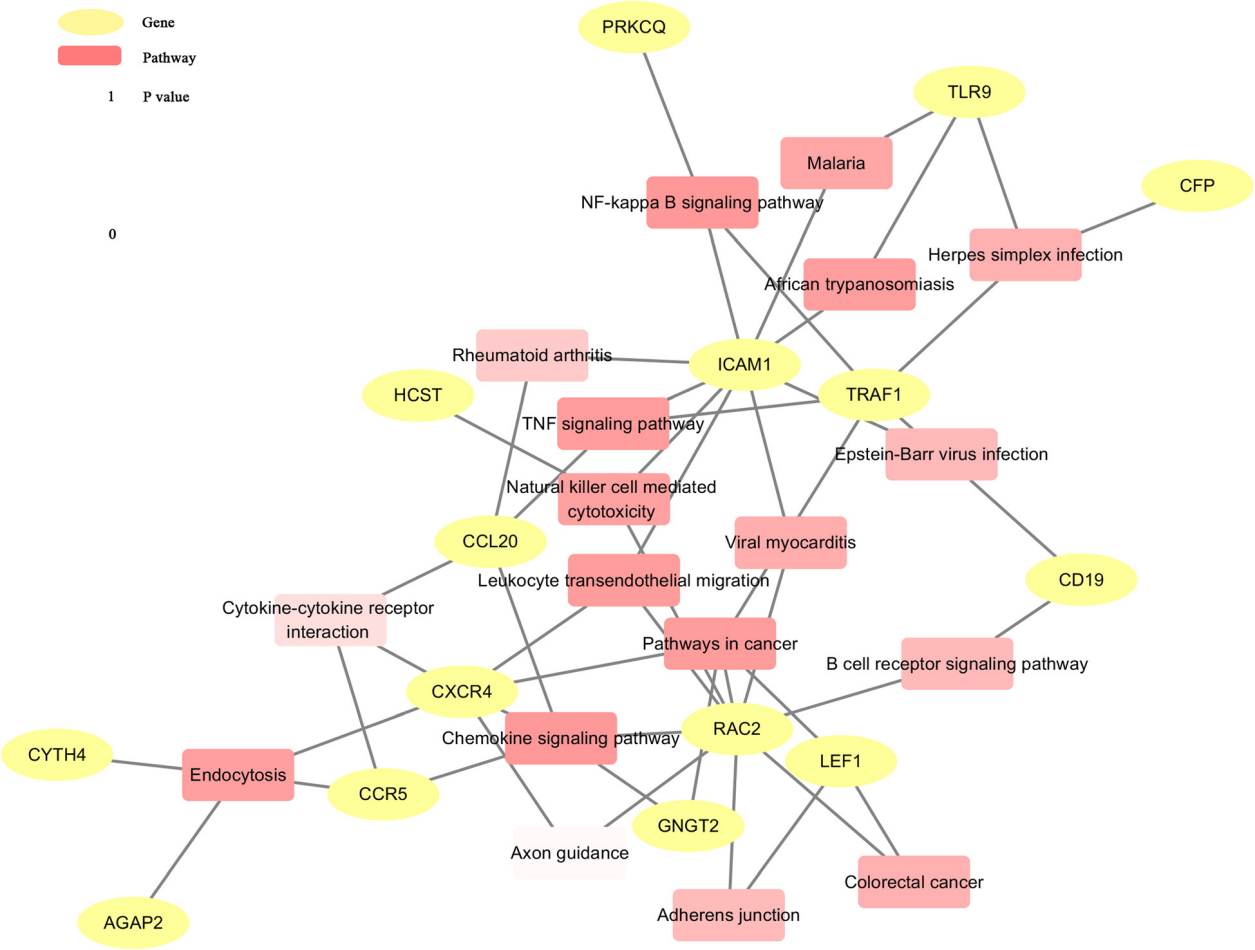
Gene-pathway network graph of DE lncRNA-related mRNAs from Table 4. *Circles* and *boxes* represent DE lncRNAs-related genes and the corresponding pathways, respectively. The color of the pathway terms is defined by the *P* value of enrichment

Twenty-eight DE mRNAs from the co-expression network data strongly correlated with the eight validated lncRNAs (*P* <0.001, *r* >0.99). Table 4 shows the detailed information of these mRNAs. Figure 6 shows that gene-pathway network graph analyses revealed that the 28 DE mRNAs, including ICAM1, TLR9, TNF receptor-associated factor 1 (TRAF1), CXCR4, chemokine (C-C motif) ligand 20 (CCL20), and CD19 were likely involved in chemokine signaling pathways, the nuclear factor-kappa B (NF-Κb) signaling pathway, TNF signaling pathway, African trypanosomiasis, natural killer cell-mediated cytotoxicity, and Epstein-Barr virus infection [27–30], which are involved in the pathogenesis of pSS. Additional file 6: Figures S1 and Additional file 7: Figure S2 show that the results of immunohistochemistry in LSGs of pSS patients confirmed the overexpression of CD19, CXCR4, ICAM1, and TLR9 proteins. These results indicate the functional roles of DE lncRNAs in the progress of pSS. Since many of these lncRNA are supposed to regulate immune functions, the correlation between the eight upregulated lncRNAs and main histopathological data, such as the infection of Epstein-Barr virus, B/T cell segregation, and presence/absence of ectopic lymphoid structures, were analyzed. The results revealed no significant correlation exists (Additional file 8: Figure S5).

**Table 4:** Detailed information of DE lncRNAs linked DE mRNA

mRNA gene symbol	*P* value	Fold change	Regulation	Correlated lncRNA gene name	*r* value	*P* value
ACY3	0.028	2.221	up	n340599	0.9996	0.0004
AGAP2	0.016	2.618	up	ENST00000420219.1, n336161	0.9995 0.9992	0.0005 0.0008
ARHGAP30	0.019	2.377	up	n336161, ENST00000455309.1	0.9993 0.9991	0.0007 0.0009
CCL20	0.013	4.295	up	TCONS_l2_00014794	0.9991	0.0009
CCR5	0.003	4.040	up	ENST00000455309.1	0.9993	0.0007
CD19	0.033	5.657	up	n340599	0.9992	0.0008
CD6	0.002	2.604	up	ENST00000420219.1	09990	0.0009
CFP	0.011	2.089	up	ENST00000546086.1, TCONS_l2_00014794	0.9995 0.9996	0.0005 0.0004
CXCR4	0.028	5.527	up	ENST00000546086.1, TCONS_l2_00014794	0.9998 0.9997	0.0002 0.0003
CYTH4	0.015	2.151	up	n340599	0.9996	0.0004
GIMAP4	0.004	2.905	up	ENST00000455309.1	0.9997	0.0003
GNGT2	0.011	3.857	up	ENST00000455309.1, n336161	0.9991 0.9999	0.0009 0.0001
HCST	0.005	3.011	up	ENST00000546086.1, TCONS_l2_00014794	0.9998 0.9995	0.0002 0.0005
HIST1H2AI	0.033	3.800	up	ENST00000455309.1	0.9995	0.0005
ICAM1	0.008	2.385	up	ENST00000455309.1 n336161	0.9991 0.9994	0.0009 0.0006
KIF20B	0.007	2.118	up	ENST00000455309.1,	0.9992	0.0008
LAPTM5	0.022	3.223	up	ENST00000546086.1	0.9997	0.0003
LEF1	0.020	3.743	up	TCONS_l2_00014794	0.9999	0.0001
LILRB1	0.003	3.180	up	ENST00000420219.1, ENST00000455309.1, n336161	0.9994 0.9998 0.9997	0.0006 0.00020.0003
PRKCQ	0.025	2.969	up	ENST00000455309.1	0.9994	0.0006
RABGAP1L	0.010	2.149	up	n340599	0.9997	0.0003
RAC2	0.006	3.169	up	n340599	0.9992	0.0008
RLTPR	0.016	2.094	up	ENST00000420219.1, n336161	0.9994 0.9993	0.0006 0.0007
SUSD3	0.010	2.587	up	ENST00000546086.1, TCONS_l2_00014794	0.9998, 0.9999	0.0002 0.0001
TLR9	0.043	2.029	up	n340599	0.9992	0.0008
TRAF1	0.014	2.530	up	n340599	0.9998	0.0002
WDFY4	0.014	3.251	up	n340599	0.9995	0.0005
ZNF831	0.014	4.663	up	ENST00000455309.1	0.9997	0.0003

*DE* differentially expressed, *lncRNA* long noncoding RNA

## Discussion

LncRNAs were primarily investigated in genomic imprinting, cancers, and cell differentiation, but these molecules are emerging as important regulators of immune cell differentiation and the activation of innate immunity. However, the identification of lncRNA expression in autoimmune diseases is largely underexplored. Few studies reported that lncRNAs played a crucial role in autoimmune diseases, such as SLE, RA, T1DM and MS [31–36], but fewer studies have examined the expression profile of lncRNAs in pSS. The critical role of lncRNAs in the pathogenesis of pSS was demonstrated previously [37]. Therefore, differentially expressed lncRNAs in the LSGs of pSS patients were identified using microarray experiments.

The results demonstrated that upregulated mRNAs were far more numerous than downregulated mRNAs in pSS samples, which indicated the activation of many new biological processes or signaling pathways in pathological conditions. DE mRNAs were further analyzed in pSS patients using GO term enrichment and pathway enrichment analyses. The GO results indicated that the most significantly enriched cellular components of upregulated mRNAs in LSGs of pSS patients were the external side of plasma membrane, immunological synapse, and MHC class II protein complex. The most significantly enriched molecular functions of upregulated mRNAs were peptide antigen binding, MHC class II receptor activity, and transmembrane signaling receptor activity. These results are consistent with previous studies that demonstrated aberrantly expressed MHC class II and costimulation molecules in the epithelial cells of salivary glands in pSS patients [38, 39]. The abnormal expression of these molecules on the surface of salivary gland epithelial cells may favor the presentation of SSA and SSB epitopes to T cells and lead to autoantibody production [40]. The immune response, inflammatory response, and cytokine-mediated signaling pathway were the most significantly enriched biological processes of the upregulated mRNAs. The results suggest that an active autoimmune inflammatory response occurred in the epithelial cells of salivary glands in pSS patients. The results also demonstrated that several cellular components, molecular functions, and biological processes were inhibited. The pathway analysis results suggested that the upregulated mRNAs in LSGs of pSS patients were significantly involved in graft-versus-host diseases, cytokine-cytokine receptor interactions, and cell adhesion molecules. The results also suggest that deficiencies occurred in the labial gland epithelial cells of pSS patients and ended with the activation of autoimmune inflammation and the release of inflammatory cytokines by immune cells.

Nine of the aberrantly expressed lncRNAs, including NR_002712, n341833, lnc-UTS2D-1:1, TCONS_l2_00014794, n336161, ENST00000420219.1, ENST00000455309.1, n340599, and ENST00000546086.1, were further confirmed in the 30 pSS patients using real-time PCR. Eight lncRNAs were significantly upregulated, but n341833 was not upregulated. Recent prospective studies confirmed that serum β2 microglobulin levels were associated with the EULAR Sjögren’s syndrome disease activity index (ESSDAI) and EULAR SS patient-reported index (ESSPRI) in the pSS patients [41, 42]. Notably, the expression levels of NR_002712, ENST00000546086.1, TCONS_l2_00014794, n340599, ENST00000455309.1, and ENST00000420219.1 correlated with the β2 microglobulin levels, which suggest that the upregulated expression of these lncRNAs was associated with active disease states. These lncRNAs may be strongly involved in the progress of pSS, especially ENST00000455309.1, which significantly correlated with the disease course, ESR, RF, and IgA expression levels. These multiple correlations were also observed with other lncRNAs. The results further confirmed that these lncRNAs played critical roles in the pathophysiology of pSS, but further functional studies are needed to examine the potential mechanisms. The regulatory mechanisms and functional principles of lncRNAs were elucidated recently. LncRNAs that regulate the abundance of genomically neighboring or distal gene products are classified as cis-regulatory models or trans-regulatory models, respectively [43]. However, lncRNAs achieve regulatory functions via modularity, the collection of diverse combinations of proteins, and possible RNA or DNA interactions. This study investigated the potential targets of eight DE lncRNAs by using an lncRNA-mRNA co-expression network. This network revealed that 28 DE mRNAs were strongly associated with eight DE lncRNAs. A gene-pathway network of the eight DE lncRNA-related genes was established to explore the functional mechanism. The results revealed a significant involvement of these eight DE lncRNAs in several important signaling pathways that play crucial roles in the pathogenesis of pSS. However, the precise regulatory mechanism of lncRNAs requires further study. Animal models should be used in further research to help elucidate the biological processes of lncRNAs in pSS. Another limitation of this study was the lack of pSS mouse models. LncRNAs expression profiles should be detected in different animal models because of the variety of pSS mouse models.

## Conclusions

This study revealed aberrant expression profiles of lncRNAs in LSGs of pSS patients for the first time. A total of 1243 lncRNAs and 1457 mRNAs were differentially expressed in the labial glands of pSS patients compared to the control subjects, and eight lncRNAs were further confirmed using real-time PCR in 30 pSS patients. Strong correlations between lncRNAs and clinical characteristics were observed. This study will lead to novel directions in pSS diagnosis and therapy.

### Ethics approval and consent to participate

This study was approved by the Ethics Committee, Faculty of Medicine, Shanghai Jiao Tong University.

## Additional files

Additional file 1: Table S1. Primer sequences used in validation of lncRNAs. (DOCX 14 kb) Additional file 2: Figure S3. Characterization of the cellular infiltrate and histomorphologic grading of LSG. (A) Shown are representative examples of grade 1 (G1; <50 periductal lymphocytes); (B,C) grade 2 (G2; >50 periductal lymphocytes): nonsegregated and segregated aggregates (NS-G2 and S-G2, respectively); (D,F) grade 3 (G3; >50 periductal lymphocytes, with GC-like structures). (TIF 58923 kb) Additional file 3: Figure S4. EBER expression in LSG of pSS patients. (A) Significant number of EBER+ cells in representative ectopic lymphoid structure-positive LSG tissue. (B) Double staining for CD3 and CD20 in the same section of LSG tissue. (C) Staining for CD21 in the same section of LSG tissue. (TIF 53011 kb) Additional file 4: Table S2. Detailed correlation analysis results. (DOCX 20 kb) Additional file 5: Table S3. Multivariate model used to identify the lncRNAs that correlated independently with disease characteristics. (DOCX 14 kb) Additional file 6: Figure S1. Immunohistochemistry for CD19 and ICAM1 in LSG of pSS and healthy control. (A, E) Infiltrating lymphocytes in LSG of pSS were positively staining for CD19 (200× and 400× magnification); (B, F) glandular epithelial cells from control were negative (200× and 400× magnification); (C, G) infiltrating lymphocytes and adjacent glandular epithelial cells in LSG of pSS were positively staining for ICAM1 (200× and 400× magnification). (D, H) Glandular epithelial cells from control were negative (200× and 400× magnification). (TIF 28780 kb) Additional file 7: Figure S2. Immunohistochemistry for TLR9 and CXCR4 in LSG of pSS and healthy controls. (A, E) Infiltrating lymphocytes in LSG of pSS positively stained for TLR9 (200× and 400× magnification); (B, F) glandular epithelial cells from control were negative (200× and 400× magnification); (C, G) infiltrating lymphocytes and adjacent glandular epithelial cells in LSG of pSS were positively stained for CXCR4 (200× and 400× magnification). (D, H) Glandular epithelial cells from control were negative (200× and 400× magnification). (TIF 28419 kb) Additional file 8: Figure S5. Expression level analysis between the eight upregulated lncRNAs and main histopathological data. (A) Expression of eight lncRNAs in grade 1 (G1), nonsegregated grade 2 (NS-G2), segregated grade 2 (S-G2), and grade 3 (G3) foci in LSG from 30 patients with pSS. (B) Expression of eight lncRNAs in EBER-positive and -negative LSG from 30 patients with pSS. (TIF 2332 kb)

## Abbreviations

C3: complement 3
C4: complement 4
CCL20: chemokine (C-C motif) ligand 20
CRP: C-reactive protein
CXCR4: C-X-C chemokine receptor type 4
DE: differentially expressed
EBER: EBV-encoded RNA
ESR: erythrocyte sedimentation rate
ESSDAI: EULAR Sjögren’s syndrome disease activity index
ESSPRI: EULAR SS patient-reported index
GO: gene ontology
ICAM1: intercellular cell adhesion molecule 1
Ig: immunoglobulin
lncRNA: long noncoding RNA
LSG: labial salivary gland
MS: multiple sclerosis
pSS: primary Sjögren’s syndrome
RA: rheumatoid arthritis
RF: rheumatoid factor
SLE: systemic lupus erythematosus
T1DM: type I diabetes mellitus
TLR9: Toll-like receptor 9
TNF-α: tumor necrosis factor alpha
TRAF1: TNF receptor-associated factor 1
VAS: visual analogue scale
