# Supplementary material for: Long non-coding RNA expression profile in minor salivary gland of primary Sjögren’s syndrome
Source: Arthritis Res Ther. 2016 May 17;18:109. doi: 10.1186/s13075-016-1005-2 (PMC4869341; doi:10.1186/s13075-016-1005-2)
Supplement: Additional file 1: Table S1. — Primer sequences used in validation of lncRNAs. (DOCX 14 kb) [file 13075_2016_1005_MOESM1_ESM.docx]

Additional file 1: Table S1. The primer sequences used in validation of LncRNAs.

| **GeneBank** | **Primer Name** | **Sequence(5'to3')** |
| --- | --- | --- |
| NR_002712 | LNCA_33_P3375859-F | CAGTGATTACCAGGGACTGAGG |
|  | LNCA_33_P3375859-R | CTGTGCCCATTAAACCATCA |
| n341833 | CUST_34915_PI429285431-F | GAGCGGAGCCTCTCCAGAA |
|  | CUST_34915_PI429285431-R | TCACAGCAGCCCCCTTTTC |
| lnc-UTS2D-1:1 | CUST_19498_PI429285431-F | CCTCCTGCATTCATTCTTCC |
|  | CUST_19498_PI429285431-R | AATCATGGTGGAAGGCAAAG |
| TCONS_l2_00014794 | CUST_48545_PI429285431-F | GACAAGCAAAGTGGATCAGCAA |
|  | CUST_48545_PI429285431-R | GAGCCAGTCTGATAGTGTTCTTTTGA |
| n336161 | CUST_30678_PI429285431-F | ATGACCAGCAAAATGCAACA |
|  | CUST_30678_PI429285431-R | CAGTTGTGAAGCGGAGATGA |
| ENST00000420219.1 | CUST_4577_PI429285431-F | TTCAATGCTTCAAACGCTCTTC |
|  | CUST_4577_PI429285431-R | GATCCCCCATTTGCATTAGCT |
| ENST00000455309.1 | CUST_1030_PI429285431-F | GGAGGCCTCAGAACATTCGA |
|  | CUST_1030_PI429285431-R | GGGTTAGGCAATCAGCAGTCA |
| n340599 | CUST_33813_PI429285431-F | AATAGCTGGCATCCTGGGAAT |
|  | CUST_33813_PI429285431-R | CCCATGAAGGCAACACCAAT |
| ENST00000546086.1 | CUST_4314_PI429285431-F | CCACTTTTTACTTATTGCAGTCCTTTC |
|  | CUST_4314_PI429285431-R | TGGGAGCAGGGAGTCATTTC |
