# Supplementary figures and images for: Long non-coding RNA expression profile in minor salivary gland of primary Sjögren’s syndrome
Source: Arthritis Res Ther. 2016 May 17;18:109. doi: 10.1186/s13075-016-1005-2 (PMC4869341; doi:10.1186/s13075-016-1005-2)

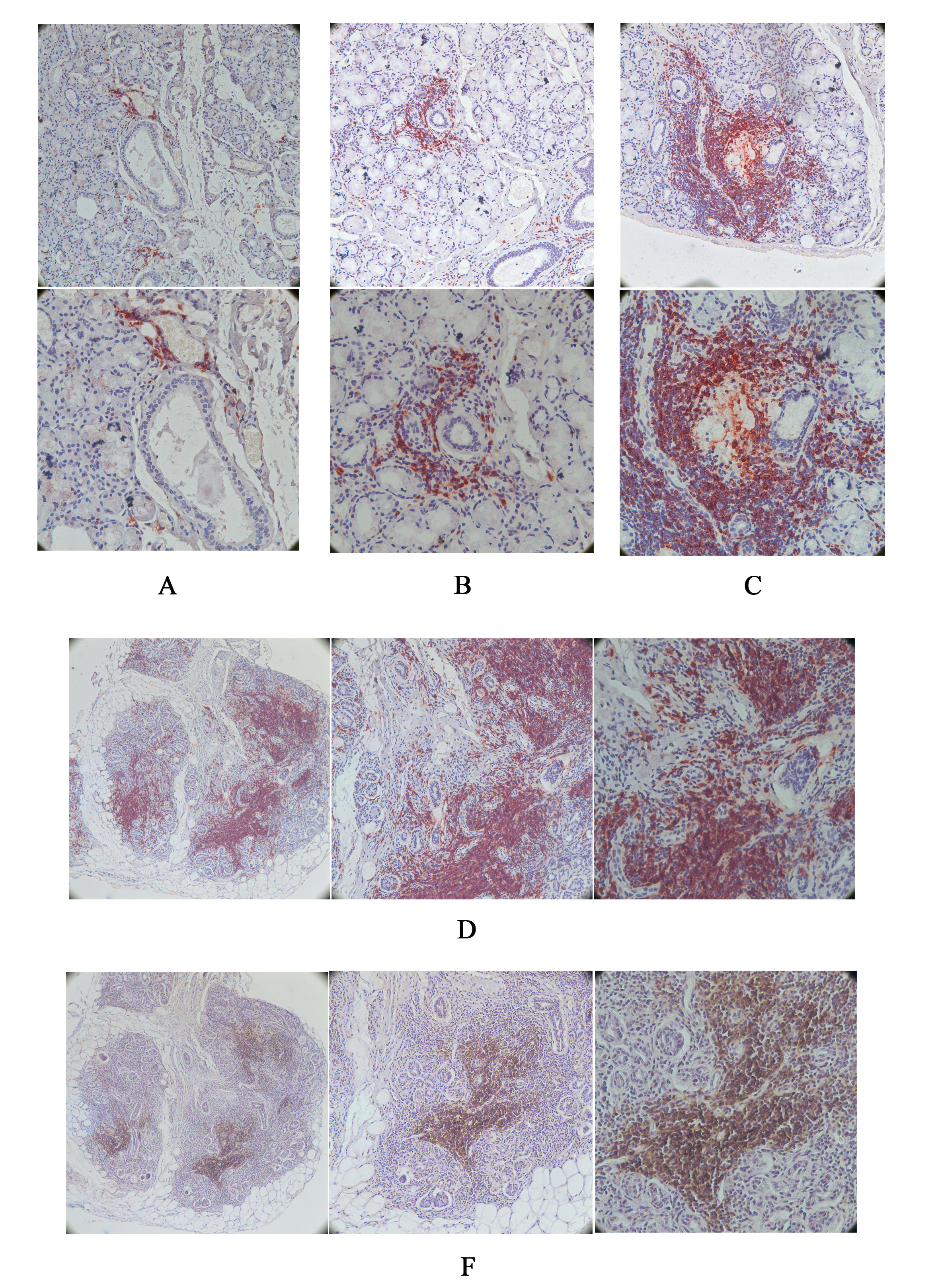

Supplement: Additional file 2: Figure S3. — Characterization of the cellular infiltrate and histomorphologic grading of LSG. (A) Shown are representative examples of grade 1 (G1; <50 periductal lymphocytes); (B,C) grade 2 (G2; >50 periductal lymphocytes): nonsegregated and segregated aggregates (NS-G2 and S-G2, respectively); (D,F) grade 3 (G3; >50 periductal lymphocytes, with GC-like structures). (TIF 58923 kb) [file 13075_2016_1005_MOESM2_ESM.tif]

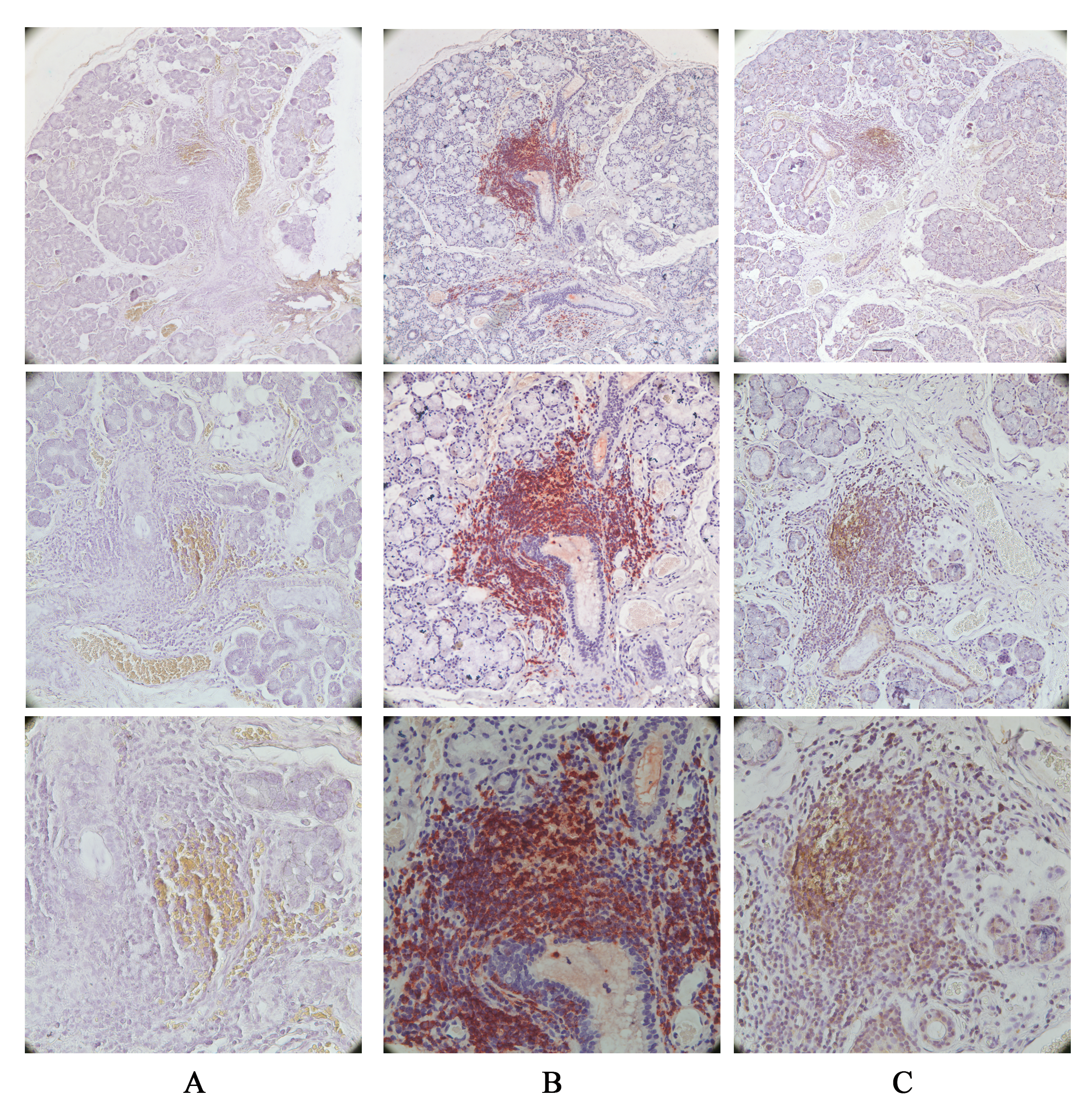

Supplement: Additional file 3: Figure S4. — EBER expression in LSG of pSS patients. (A) Significant number of EBER+ cells in representative ectopic lymphoid structure-positive LSG tissue. (B) Double staining for CD3 and CD20 in the same section of LSG tissue. (C) Staining for CD21 in the same section of LSG tissue. (TIF 53011 kb) [file 13075_2016_1005_MOESM3_ESM.tif]

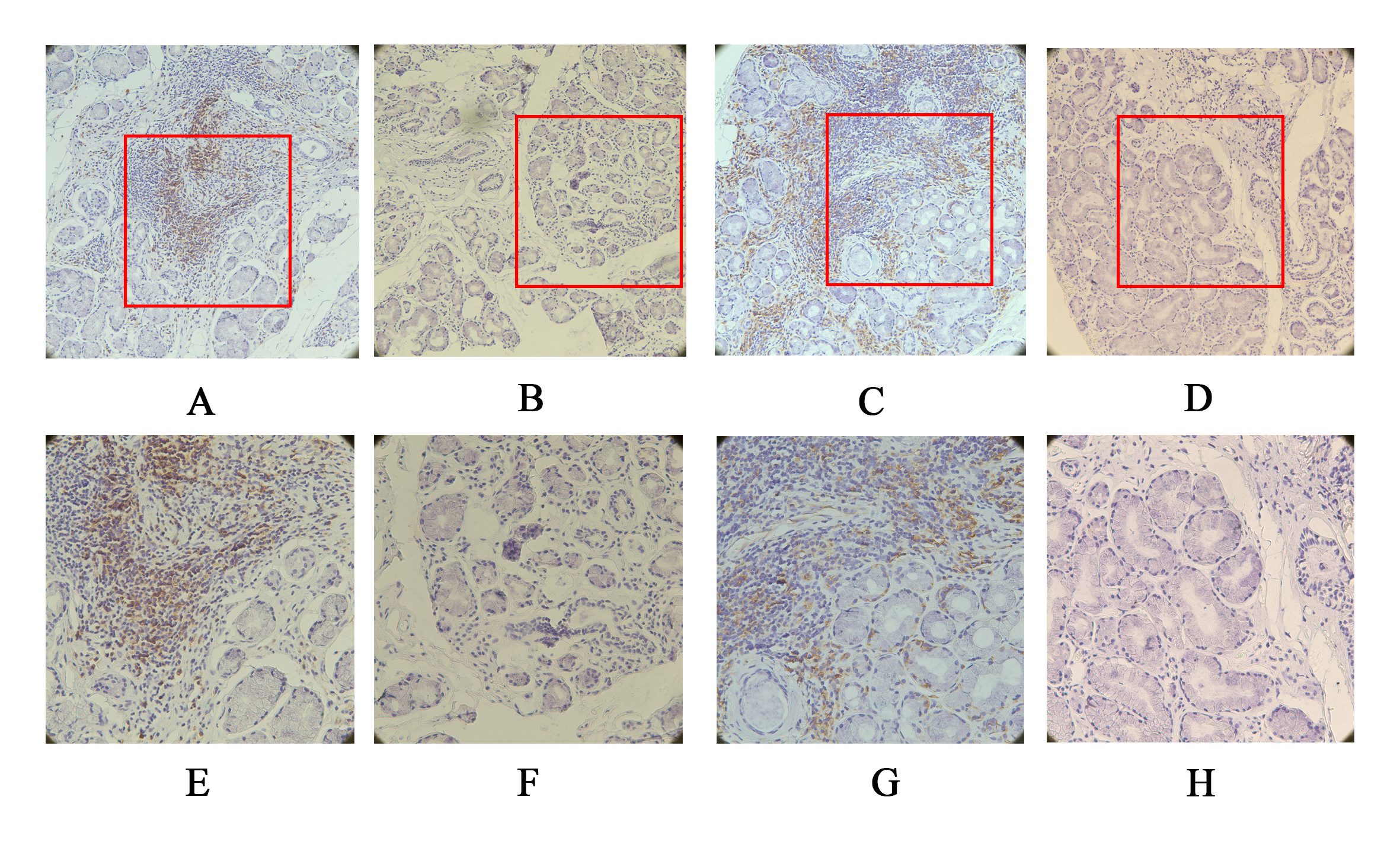

Supplement: Additional file 6: Figure S1. — Immunohistochemistry for CD19 and ICAM1 in LSG of pSS and healthy control. (A, E) Infiltrating lymphocytes in LSG of pSS were positively staining for CD19 (200× and 400× magnification); (B, F) glandular epithelial cells from control were negative (200× and 400× magnification); (C, G) infiltrating lymphocytes and adjacent glandular epithelial cells in LSG of pSS were positively staining for ICAM1 (200× and 400× magnification). (D, H) Glandular epithelial cells from control were negative (200× and 400× magnification). (TIF 28780 kb) [file 13075_2016_1005_MOESM6_ESM.tif]

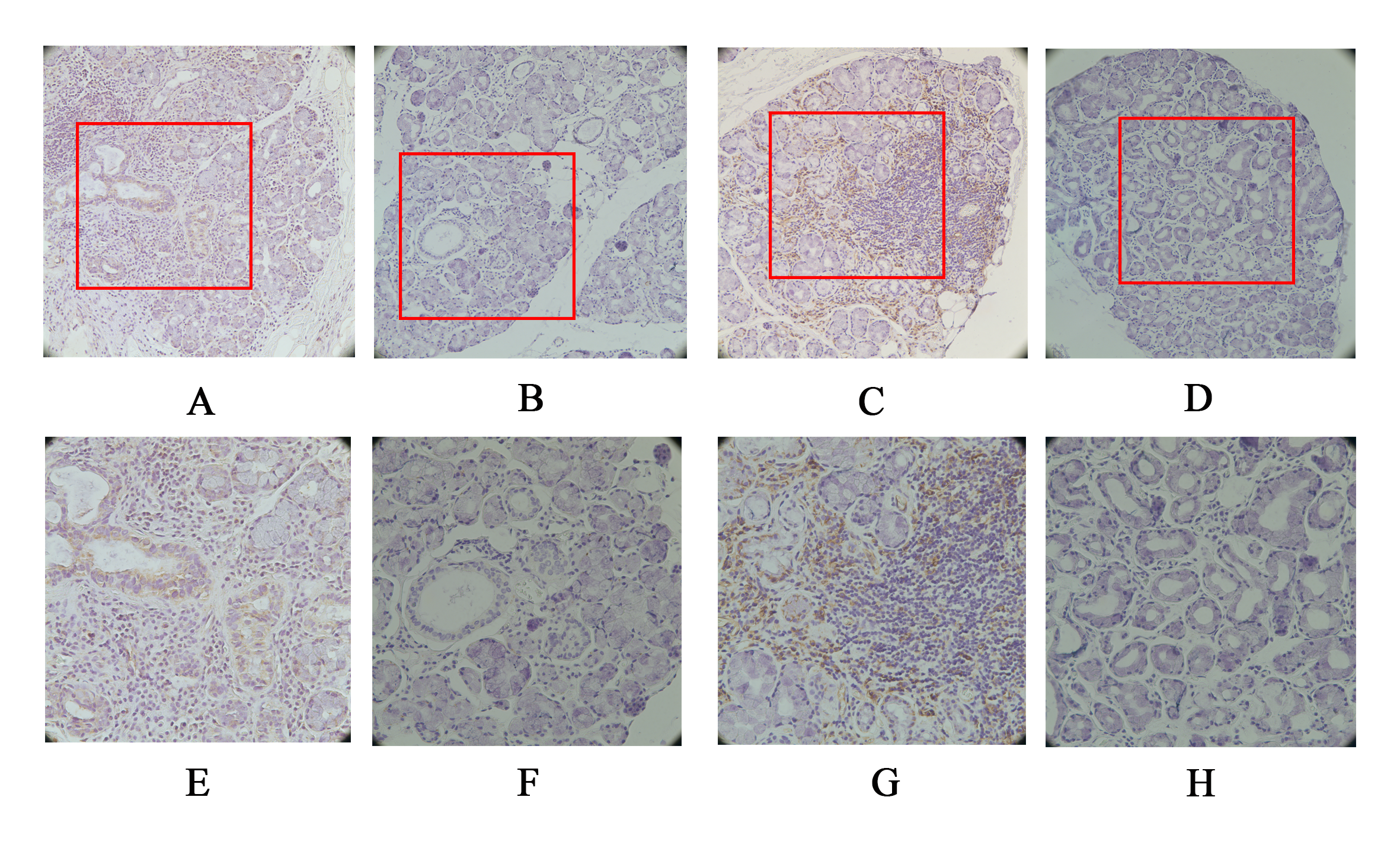

Supplement: Additional file 7: Figure S2. — Immunohistochemistry for TLR9 and CXCR4 in LSG of pSS and healthy controls. (A, E) Infiltrating lymphocytes in LSG of pSS positively stained for TLR9 (200× and 400× magnification); (B, F) glandular epithelial cells from control were negative (200× and 400× magnification); (C, G) infiltrating lymphocytes and adjacent glandular epithelial cells in LSG of pSS were positively stained for CXCR4 (200× and 400× magnification). (D, H) Glandular epithelial cells from control were negative (200× and 400× magnification). (TIF 28419 kb) [file 13075_2016_1005_MOESM7_ESM.tif]

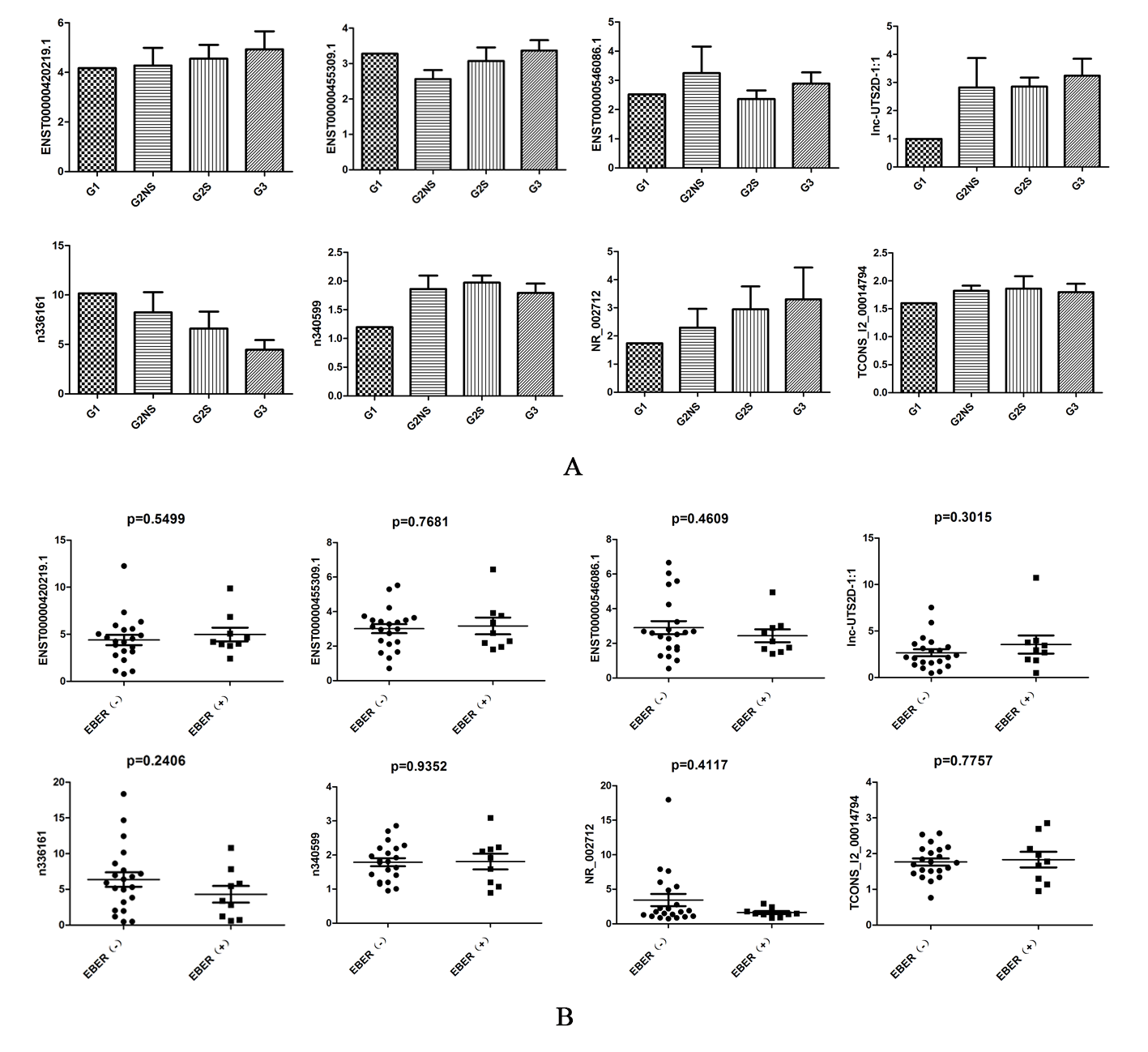

Supplement: Additional file 8: Figure S5. — Expression level analysis between the eight upregulated lncRNAs and main histopathological data. (A) Expression of eight lncRNAs in grade 1 (G1), nonsegregated grade 2 (NS-G2), segregated grade 2 (S-G2), and grade 3 (G3) foci in LSG from 30 patients with pSS. (B) Expression of eight lncRNAs in EBER-positive and -negative LSG from 30 patients with pSS. (TIF 2332 kb) [file 13075_2016_1005_MOESM8_ESM.tif]
