# Supplementary material for: Long non-coding RNA expression profile in minor salivary gland of primary Sjögren’s syndrome
Source: Arthritis Res Ther. 2016 May 17;18:109. doi: 10.1186/s13075-016-1005-2 (PMC4869341; doi:10.1186/s13075-016-1005-2)
Supplement: Additional file 4: Table S2. — Detailed correlation analysis results. (DOCX 20 kb) [file 13075_2016_1005_MOESM4_ESM.docx]

Additional file 4: Table S2. The detailed correlation analysis results.

| LncRNA | VAS of dry mouth | VAS of dry eyes | VAS of parotid swelling | Saxon text | Disease course | ESR | RF | β2 microglobulin | IgG | IgM |
| --- | --- | --- | --- | --- | --- | --- | --- | --- | --- | --- |
| ENST00000420219.1 | P=0.6642  R=0.0826 | P=0.1706  R=0.2569 | P=0.0127  R=0.4493 | P=0.5293  R=-0.1995 | P=0.0033  R=0.5184 | P=0.1609  R=0.2626 | P=0.3612  R=0.1728 | P=0.0346  R=0.4006 | P=0.1980  R=0.2418 | P=0.2391  R=0.2217 |
| ENST00000455309.1 | P=0.3811  R=-0.1659 | P=0.2938  R=0.1982 | P=0.4935  R=0.1300 | P=0.1179  R=0.2916 | P=0.0292  R=0.3984 | P=0.0164  R=0.4345 | P=0.0059  R=0.4905 | P=0.0231  R=0.4279 | P=0.5436  R=0.1154 | P=0.7846  R=0.0521 |
| ENST00000546086.1 | P=0.5364  R=-0.1175 | P=0.1886  R=0.2468 | P=0.2165  R=0.2324 | P=0.3741  R=0.1638 | P=0.0307  R=0.3952 | P=0.0667  R=0.3392 | P=0.0058  R=0.4918 | P=0.0024  R=0.5508 | P=0.5802  R=0.1052 | P=0.7617  R=0.0577 |
| Lnc-UTS2D-1:1 | P=0.9491  R=-0.0122 | P=0.7321  R=0.0652 | P=0.0833  R=0.3214 | P=0.7856  R=0.0518 | P=0.0728  R=0.3323 | P=0.6005  R=0.0996 | P=0.1719  R=0.2561 | P=0.4304  R=0.1552 | P=0.7339  R=0.0648 | P=0.7569  R=0.0590 |
| n336161 | P=0.3087  R=0.1923 | P=0.0278  R=0.4016 | P=0.9793  R=0.0050 | P=0.6998  R=0.0734 | P=0.0352  R=0.3858 | P=0.0356  R=0.3850 | P=0.1711  R=0.2566 | P=0.1012  R=0.3161 | P=0.7972  R=-0.0490 | P=0.0714  R=-0.3338 |
| n340599 | P=0.9515  R=0.0120 | P=0.6009  R=0.0995 | P=0.1479  R=0.2696 | P=0.7163  R=-0.0692 | P=0.0002  R=0.6258 | P=0.2186  R=0.2458 | P=0.8908  R=0.0262 | P=0.0191  R=0.4400 | P=0.2014  R=0.2400 | P=0.2763  R=0.2053 |
| NR_002712 | P=0.9326  R=-0.0161 | P=0.3113  R=0.1913 | P=0.0280  R=0.4001 | P=0.2049  R=-0.2382 | P=0.0690  R=0.3322 | P=0.0131  R=0.4479 | P=0.0538  R=0.3556 | P=0.0002  R=0.6452 | P=0.1122  R=0.2960 | P=0.0490  R=0.3625 |
| TCONS_l2_00014794 | P=0.6681  R=-0.0816 | P=0.4414  R=0.1460 | P=0.1331  R=0.2806 | P=0.1688  R=-0.3725 | P=0.0156  R=0.4367 | P=0.3079  R=0.0979 | P=0.3689  R=0.1701 | P=0.0034  R=0.5339 | P=0.2735  R=0.2065 | P=0.8720  R=0.0307 |

| LncRNA | IgA | IgE | CRP | C3 | C4 | Grading of labial biopsy |
| --- | --- | --- | --- | --- | --- | --- |
| ENST00000420219.1 | P=0.0099  R=0.4632 | P=0.2226  R=0.2294 | P=0.9373  R=-0.0159 | P=0.8485  R=-0.0378 | P=0.9873  R=-0.0032 | P=0.3475  R=0.1777 |
| ENST00000455309.1 | P=0.0085  R=0.4716 | P=0.9930  R=0.0016 | P=0.6005  R=0.1055 | P=0.6029  R=0.1027 | P=0.4834  R=0.1330 | P=0.0856  R=0.3191 |
| ENST00000546086.1 | P=0.0030  R=0.5238 | P=0.3856  R=0.1643 | P=0.9821  R=-0.0045 | P=0.7167  R=-0.0718 | P=0.2736  R=0.2185 | P=0.3757  R=0.1677 |
| Lnc-UTS2D-1:1 | P=0.3998  R=0.1595 | P=0.6006  R=0.0995 | P=0.8574  R=-0.0362 | P=0.1709  R=-0.2662 | P=0.8581  R=-0.0361 | P=0.1448  R=0.2727 |
| n336161 | P=0.2486  R=-0.2174 | P=0.4792  R=-0.1343 | P=0.6773  R=0.0839 | P=0.0493  R=-0.3705 | P=0.3465  R=-0.1885 | P=0.0626  R=-0.3441 |
| n340599 | P=0.9275  R=0.0174 | P=0.2398  R=0.2213 | P=0.2210  R=0.2435 | P=0.8768  R=-0.0306 | P=0.3184  R=-0.1995 | P=0.6159  R=0.0954 |
| NR_002712 | P=0.4577  R=0.1490 | P=0.4507  R=-0.1431 | P=0.1091  R=0.3145 | P=0.4134  R=0.1609 | P=0.5613  R=0.1169 | P=0.8870  R=-0.0295 |
| TCONS_l2_00014794 | P=0.6450  R=0.0877 | P=0.1804  R=0.2513 | P=0.9552  R=-0.0113 | P=0.2730  R=-0.2145 | P=0.3424  R=-0.1576 | P=0.9084  R=0.0219 |
