# Supplementary material for: Long non-coding RNA expression profile in minor salivary gland of primary Sjögren’s syndrome
Source: Arthritis Res Ther. 2016 May 17;18:109. doi: 10.1186/s13075-016-1005-2 (PMC4869341; doi:10.1186/s13075-016-1005-2)
Supplement: Additional file 5: Table S3. — Multivariate model used to identify the lncRNAs that correlated independently with disease characteristics. (DOCX 14 kb) [file 13075_2016_1005_MOESM5_ESM.docx]

Additional file 5: Table S3. The multivariate model to identify the lncRNAs correlate independently with disease characteristics.

| Y value（stepwise） | X value  (lncRNA) | β | SE | Standardized β | t | P |
| --- | --- | --- | --- | --- | --- | --- |
| β2 microglobulin | TCONS_l2_00014794 | 1.489 | 0.542 | 0.461 | 2.749 | 0.010 |
| ESR | NR_002712 | 6.721 | 2.297 | 0.484 | 2.926 | 0.007 |
| Course of disease | n340599 | 43.267 | 11.842 | 0.568 | 3.654 | 0.001 |
| IgA | ENST00000546086.1 | 0.508 | 0.216 | 0.406 | 2.350 | 0.026 |
| VAS of parotid swelling | NR_002712 | 1.371 | 0.417 | 0.527 | 3.284 | 0.003 |
| VAS of dry eyes | n336161 | 0.877 | 0.400 | 0.383 | 2.191 | 0.037 |
| IgE | ENST00000455309.1 | -143.183 | 46.184 | -1.026 | -3.100 | 0.005 |
|  | ENST00000546086.1 | 136.678 | 42.197 | 1.222 | 3.239 | 0.004 |
